# Effect of *Spirulina maxima* Supplementation on Calcium, Magnesium, Iron, and Zinc Status in Obese Patients with Treated Hypertension

**DOI:** 10.1007/s12011-016-0623-5

**Published:** 2016-01-16

**Authors:** J. Suliburska, M. Szulińska, A. A. Tinkov, P. Bogdański

**Affiliations:** 1 Department of Human Nutrition and Hygiene, Poznan University of Life Sciences, Wojska Polskiego 31 Str., 60-624 Poznan, Poland; 2 Department of Education and Obesity Treatment and Metabolic Disorders, University of Medical Sciences, Poznan, Poland; 3 Orenburg State Medical University, Orenburg, Russia; 4 All-Russian Research Institute of Medicinal and Aromatic Plants (VILAR), Moscow, Russia

**Keywords:** Spirulina, Obesity, Treated hypertension, Minerals, Randomized trial

## Abstract

The effects of *Spirulina maxima* supplementation on calcium, magnesium, iron, and zinc status were studied in a double-blind placebo-controlled trial of 50 obese subjects with treated hypertension, each randomized to receive 2 g of spirulina or a placebo daily for 3 months. At baseline and after treatment, the calcium, magnesium, iron, and zinc concentration in plasma was assessed. It was found that 3 months of *S*. *maxima* supplementation resulted in a significant decrease in the iron level in the plasma of obese patients. In conclusion, this is the first clinical study on the influence of spirulina supplementation on mineral status in obese patients with hypertension. Spirulina supplementation affects the iron status of obese Caucasians with well-treated hypertension.

## Introduction

Obesity is associated with an impairment of the body’s mineral status. Multiple studies have indicated that obesity is accompanied by altered iron homeostasis [1]. At the same time, weight reduction has been associated with improved iron metabolism [2]. It has been demonstrated that serum magnesium content decreases in experimental [3] and clinical studies [4, 5]. Moreover, it has been proposed that magnesium deficiency in obese subjects may contribute to hypertension [6] and insulin resistance [5]. Recent data also indicate an association between inadequate calcium intake and increased body weight [7]. It has been also proposed that increased consumption of calcium in the diet may result in improvements in obesity and hypertension [8]. The mechanisms of antiobesity action of dietary calcium may involve decreased fat absorption, regulation of adipocyte proliferation, and apoptosis [9]. Finally, zinc concentration in both plasma and the erythrocytes of obese individuals was significantly lower than in lean individuals [10]. Experimental studies also provide additional data on the role of zinc deficiency in obesity pathogenesis in high-fat fed mice [11]. Taking into account the possible role of impaired zinc balance in hypertension [12], obesity-associated zinc deficiency may be an additional link between these two pathologies. It was found in another study that improvements in zinc status were associated with increases in insulin sensitivity in obese patients [13, 14].

Blue-green freshwater algae may be a good source of minerals for obese people, and especially of calcium and iron, due to their substantial levels [15, 16]. Moreover, green algae show promising antiobesity properties. Spirulina is widely used in functional nutrition and medicine due to its antioxidant, anti-inflammatory, anticancer, and immunostimulatory activities [17]. At the same time, these microalgae are able to accumulate significant amounts of trace elements, thus serving as a supplemental nutritional source of essential elements [18]. Moreover, the metal-accumulating ability of spirulina is used in engineering to enrich it with certain trace elements [19].

The hypothesis in the present study is that oral supplementation with spirulina has an influence on mineral status in obese hypertensive patients receiving standard antihypertensive treatment. Patients were therefore randomized to receive 2 g of *Spirulina maxima* or a matching placebo daily, in order to measure the concentration of calcium, magnesium, iron, and zinc in plasma at baseline and after 3 months of treatment.

## Materials and Methods

### Study Patients

The study protocol was approved by the Research Ethics Committee of Poznań University of Medical Sciences, registered as case no. 599/12. Informed consent was obtained from all patients. The study was performed in accordance with the Declaration of Helsinki.

Among the 142 registered patients with hypertension and obesity screened in our outpatient clinic, a total of 50 (25 men, 25 women) were enrolled to the study.

The inclusion criteria were body mass index (BMI) equal to or greater than 30 kg/m^2^, age 25 to 60 years, stable body weight (<3 kg self-reported change during the previous 3 months), and well-controlled hypertension (meaning systolic blood pressure (SBP) less than 160 mmHg and diastolic blood pressure (DBP) less than 100 mmHg) with stable treatment for at least 6 months.

The exclusion criteria were secondary obesity or secondary hypertension; diabetes; a history of coronary artery disease, stroke, congestive heart failure, or malignancy; a history of the use of any dietary supplements within the 3 months prior to the study; a current need for modification of antihypertensive therapy; abnormal liver or kidney function; any clinically significant process; a history of infection in the month prior to the study; nicotine or alcohol abuse; or other condition that, in the opinion of the investigators, would make participation not in the best interest of the patient or could prevent, limit, or confound the protocol-specified efficacy assessments.

### Study Design

The study was designed as a randomized double-blind placebo-controlled trial with two parallel groups. Randomization was performed by an independent statistician. The participants were randomly assigned (in a 1:1 ratio) to receive with their morning meal for 3 months four capsules of either Hawaiian spirulina (Cyanotech Corporation, HI, USA) or a placebo. All supplements were packed in bottles without labeling. The Hawaiian spirulina capsules each contained 0.5 g of *S*. *maxima* (60–70 % protein, gamma-linolenic acid (GLA), beta carotene, iron, and phycocyanin (PC)). The placebo consisted of pure microcrystalline cellulose. All patients were advised to continue their habitual diet and exercise patterns throughout the study. The intention-to-treat (ITT) population consisted of 50 patients.

### Anthropometric and Blood Pressure Measurements

During the anthropometric measurements, the patients wore lightweight clothing and no shoes. Weight was measured to the nearest 0.1 kg and height to the nearest 0.1 cm. Obesity was defined as a BMI of 30 kg/m^2^ or greater (WHO, 1995). Blood pressure was measured seated, according to the guidelines of the European Society of Hypertension [20], using a digital electronic tensiometer (model 705IT, Omron Corporation, Kyoto, Japan). Hypertension was defined as the average of three measurements of arterial blood pressure obtained after 10 min of physical rest by the patients (twice at two different visits in the same month).

### Biochemical Measurements

Blood samples were taken from each participant following an overnight fast and after lying in a supine position for 30 min.

The levels of calcium, magnesium, iron, and zinc in plasma were determined by flame atomic absorption spectrometry (with a Zeiss AAS-3 spectrometer with deuterium background correction). In order to obtain the concentration of the plasma elements, the samples were diluted (*v*/*v* 1:1) as follows: for iron and zinc analyses, 0.01 % Triton X-100 (Merck) was used, while for the calcium and magnesium analysis, aqueous solutions consisting of 0.01 % Triton X-100 (Merck) and 0.05 % lanthanum chloride (Merck) were used. The amounts of iron, zinc, calcium, and magnesium in the plasma samples were determined at the following respective wavelengths: 248.3, 213.9, 422.7, and 285.2 nm. The accuracy of the method was verified using a certified reference material (HUM ASY CONTROL 2, Randox) and was 95, 99, 94, and 99 % for calcium, magnesium, iron, and zinc, respectively.

### Dietary and Supplement Intake

Every 14 days, and also 3 days before the laboratory tests, dietary intake was assessed on the basis of dietary intake interviews (24-h recall). The level of nutrients in the daily diet was evaluated using a dietetics computer program (Dieta 5.0, 2011). The intake of nutrients and caffeine consumption during the study were constant and comparable between the groups. Physical activity was constant and unchanged in all subjects at the beginning of and after treatment.

In this study, a single daily dosage of supplement was used in order to facilitate the patients taking it. During the interview with each patient on the first day of the study, the physician described taking the daily spirulina supplement and its benefits. Every 14 days, the patient visited the dietician to record the number of pills consumed over the previous 2-week period. During the first visit to the dietician, each patient received a diary in which to enter the time the supplement was taken each day. On subsequent visits, the diary was checked by either a dietician or a physician. The investigators maintained a log of all pills dispensed and returned. The drugs supplied to each subject were accounted for throughout the study. The level of compliance required was 90 %.

## Statistical Analysis

It was calculated that a sample size of at least 20 patients in each group would yield at least an 80 % chance of detecting a treatment effect as being statistically significant at the 0.05 alpha level. The results are given as mean values. Statistical calculations were performed using Statistica 10.0 software (StatSoft, Inc., Tulsa, OK, USA). The normality test was performed to assess normal distribution within the data. Statistical analysis was carried out using a factorial ANOVA. Mann-Whitney *U* test was used to assess the differences between the groups at baseline. ITT analysis was performed. A *p* value of less than 0.05 was regarded as significant.

## Results

The baseline characteristics of both groups are shown in Table 1. There were no statistically significant differences between the two treatment groups prior to the study. All subjects completed the study, and no significant changes in diets, physical activity, or antihypertensive treatment were recorded.

**Table 1:** Baseline characteristics of Spirulina group and Placebo group (value are means ± SD)

Analyzed parameters	Spirulina group (*n* = 25)	Placebo group (*n* = 25)	*P* ^1^
Male (*n*)	12	13	
Female (*n*)	13	12	
Age (year)	49.3 ± 8.7	50.2 ± 7.2	NS
Time since diagnosis of hypertension (year)	5.7 ± 2.1	5.6 ± 2.5	NS
Medication (*n*)			
ACEI	12	11	
Sartan	2	4	
CCB	9	8	
Β-bloker	4	3	
Diuretic	7	6	
BMI (kg/m^2^)	33.5 ± 6.7	33.3 ± 6.2	NS
SBP (mmHg)	148 ± 15	151 ± 15	NS
DBP (mmHg)	84 ± 9	85. ± 9	NS
Ca (mmol/L)	3.4 ± 0.3	3.3 ± 0.2	NS
Mg (mmol/L)	0.8 ± 0.1	0.8 ± 0.1	NS
Fe (μmol/L)	16.6 ± 3.8	15.8 ± 2.6	NS
Zn (μmol/L)	10.0 ± 3.5	10.7 ± 2.5	NS

*ACEI* angiotensin-converting enzyme inhibitor, *CCB* calcium channel blocker, *NS* not statistically significant, *BMI* body mass index, *SBP* systolic blood pressure, *DBP* diastolic blood pressure, *SD* standard deviation

^1^Differences were tested using Mann-Whitney *U* test

The amount of minerals in *S*. *maxima* is shown in Table 2.

**Table 2:** Concentration of minerals in Hawaiian spirulina supplement

Minerals	In 100 g of powder	In 2 g (4 capsules) of powder
Ca (mg)	583.3	11.7
Mg (mg)	746.7	14.9
Fe (mg)	175.3	3.5
Zn (mg)	57.3	1.2

The values of the concentration of the minerals in plasma in the spirulina and placebo groups, both before and after treatment, are summarized in Table 3, along with the *p* values for the “treatment” and “time” factors and their interactions, as obtained from the ANOVA analysis. A statistically significant interaction between treatment and time factors (with both factors being statistically significant) was observed only for the iron level. The iron level was found to be significantly lower in the spirulina group than in the placebo group.

**Table 3:** Changes in mineral status during the supplementation in the spirulina and placebo groups

Analyzed parameters		Baseline		After 3 months		ANOVA		
		Spirulina group (*n* = 25)	Placebo group (*n* = 25)	Spirulina group (*n* = 25)	Placebo group (*n* = 25)	Treatment *P* value	Time	Interaction
Intention-to-treat population, *n* = 50								
Ca (mmo/L)	Mean ± SD	3.41 ± 0.27	3.25 ± 0.20	3.55 ± 0.32	3.31 ± 0.33	0.202	0.106	0.122
	Median	3.43	3.27	3.53	3.29			
Mg (mmol/L)	Mean ± SD	0.75 ± 0.07	0.80 ± 0.11	0.75 ± 0.10	0.78 ± 0.09	0.288	0.203	0.102
	Median	0.74	0.78	0.75	0.79			
Fe (μmol/L)	Mean ± SD	16.63 ± 3.75	15.84 ± 2.55	12.98 ± 2.75	16.11 ± 3.12	*0.008*	*0.008*	*0.001*
	Median	16.58	15.90	13.75	15.98			
Zn (μmol/L)	Mean ± SD	10.03 ± 3.54	10.71 ± 2.50	9.78 ± 2.14	10.92 ± 1.81	0.517	0.105	0.418
	Median	9.09	9.30	9.36	9.38			

Data are arithmetic mean ± SD

*SD* standard deviation, *NS* not significant

Italic values indicate ANOVA test, significant differences at *P* < .05

Following treatment, the concentration of calcium, magnesium, and zinc in plasma was not changed markedly in either of the groups.

## Discussion

The data indicate that spirulina consumption resulted in significantly decreased serum iron concentrations. Hypothetically, this effect may occur as a result of the iron-chelating activity of spirulina. In particular, it has been demonstrated that phycocyanin isolated from *Spirulina platensis* extract is capable of binding ferrous ions (from FeSO_4_) and ferric ions (from FeCl_3_) [21]. The presence of phycocyanin in other spirulina species, including the *S*. *maxima* [22] used in the current study, supports this hypothesis. Furthermore, it is known that blue-green algae bioaccumulate heavy metals and they act as bioadsorbents for them [23, 24]. Therefore, spirulina may decrease absorption of iron from food. Moreover, lower bioavailability of iron may be partly caused by interaction between heavy metals in spirulina and iron, because as it is known, e.g., cadmium inhibits iron absorption.

There is a growing body of data on the influence of spirulina on metabolic syndrome and its components. In particular, consumption of spirulina in a mouse model of metabolic syndrome based on monosodium glutamate injection resulted in decreased serum lipids and leptin concentrations. Moreover, spirulina has been shown to reduce liver and adipose tissue inflammation, and even more effective than pioglitazone [25]. Similar results have been observed in a recent study [26]. The earlier study also demonstrated a protective effect of spirulina against fructose-induced fatty liver formation [27]. Existing clinical studies conform to the experimental data. It has been shown that the intake of 2.8 g of spirulina thrice a day results in a significant reduction in body weight [28]. A report on three cases of nonalcoholic fatty liver disease indicated the efficacy of *S*. *maxima* consumption in improving metabolic and ultrasonographic parameters [29]. *S*. *maxima* is also considered a potential antiobesity drug [30]. Taking into account the role of oxidative stress and inflammation in the pathogenesis of obesity and metabolic syndrome, the antioxidant and anti-inflammatory properties of spirulina [31] may mediate the observed protective effect.

A number of studies have highlighted the role of impaired iron homeostasis in metabolic syndrome [32] and its components, such as obesity [33] and nonalcoholic fatty liver disease [34]. Moreover, iron chelation has been shown to be efficient in the prevention of adipocyte hypertrophy, oxidative stress, and inflammation [35]. In previous studies, a deficit of iron in western diet, high in fat and sugar, improves lipid and carbohydrate metabolism [36]. In clinical trial, it was found that phlebotomy, with consecutive reduction of body iron stores, lowered blood pressure and resulted in improvements in markers of cardiovascular risk and glycemic control in patients with metabolic syndrome [37]. It is claimed that iron depletion upregulates glucose uptake and increases insulin receptor activity in hepatocytes and that the main mechanisms responsible for improving carbohydrate metabolism are associated with the inhibition of oxidative stress [36]. In the light of the recent finding, it may be supposed that the observed protective effect of spirulina in metabolic syndrome and obesity may be mediated by the improvement of iron homeostasis and the prevention of iron overload.

Unquestionably, this study has some limitations: the study group is rather small and only selected minerals were determined. In addition, the minerals were measured only in serum and the concentrations of mineral in erythrocytes or urine were not assessed. Moreover, the association between mineral status and body mass, blood pressure, and other biochemical parameters was not determined. The evaluation of these relationships would allow a wider discussion on the impact of spirulina treatment on the mineral status in obesity and hypertension.

In conclusion, treatment with *S*. *maxima* resulted in decreased serum iron levels in hypertensive obese adults. However, further in vivo and in vitro studies are required to estimate the exact mechanisms of the influence of spirulina consumption on iron homeostasis in obesity.
